# Polyphosphate - an ancient energy source and active metabolic regulator

**DOI:** 10.1186/1475-2859-10-63

**Published:** 2011-08-04

**Authors:** Lucia Achbergerová, Jozef Nahálka

**Affiliations:** 1 Slovak Academy of Sciences, Institute of Chemistry, Centre for Glycomics, Dúbravská cesta 9, SK-845 38 Bratislava, Slovakia; 2 Slovak Academy of Sciences, Institute of Chemistry, Centre of excellence for white-green biotechnology, Trieda A. Hlinku 2, SK-949 76 Nitra, Slovakia

## Abstract

There are a several molecules on Earth that effectively store energy within their covalent bonds, and one of these energy-rich molecules is polyphosphate. In microbial cells, polyphosphate granules are synthesised for both energy and phosphate storage and are degraded to produce nucleotide triphosphate or phosphate. Energy released from these energetic carriers is used by the cell for production of all vital molecules such as amino acids, nucleobases, sugars and lipids. Polyphosphate chains directly regulate some processes in the cell and are used as phosphate donors in gene regulation. These two processes, energetic metabolism and regulation, are orchestrated by polyphosphate kinases. Polyphosphate kinases (PPKs) can currently be categorized into three groups (PPK1, PPK2 and PPK3) according their functionality; they can also be divided into three groups according their homology (*Ec*PPK1, *Pa*PPK2 and *Sc*VTC). This review discusses historical information, similarities and differences, biochemical characteristics, roles in stress response regulation and possible applications in the biotechnology industry of these enzymes. At the end of the review, a hypothesis is discussed in view of synthetic biology applications that states polyphosphate and calcium-rich organelles have endosymbiotic origins from ancient protocells that metabolized polyphosphate.

## Introduction - polyP origins

**Figure 1:**
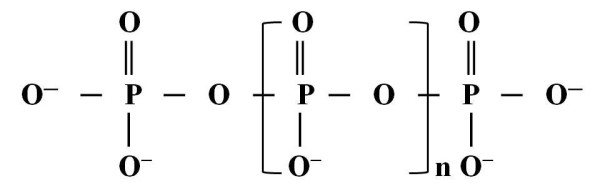
**The polyphosphate molecule**.

The first law of thermodynamics states that energy is neither created nor destroyed but can be converted from one form to another. Biological systems are beautiful models of this law in which the energy transformed into chemical potential energy is stored in covalent bonds between atoms. Later, potential energy, released by breaking certain chemical bonds, is used for biological reactions [1]. Inorganic polyphosphate (polyP) is a rich source of energy. PolyP compounds are linear polymers containing tens to hundreds of phosphate residues linked by energy-rich phosphoanhydride bonds (Figure 1) [2].

PolyP appears to have always been an easy and rich source of energy from prebiotic times to today. Unfortunately, no abiotic polyP minerals can be found on Earth today. However, some calcium pyrophosphate has been found in New Jersey and small amounts of pyrophosphate and tripolyphosphate have been found in fumaroles near Mount Usa in Hokkaido, Japan [3]. PolyP has also been found in other areas, such as the polyP found in deep oceanic steam that is a biogenic amorphous mineral. Those polyP compounds composed of calcium orthophosphates are produced from the exoskeleton structures of dead plankton [4]. For this reason, some authors think that polyP-like matter is produced only through an organism-mediated process, and so its abiotic origin in marine environments is unlikely [5]. Despite the fact that today's marine polyP has a biotic origin, one would agree with Kornberg's theory that polyP represents a "bioenergy fossil". It is a prominent energy precursor in prebiotic evolution [6] if the following three points are considered:

(i) First, pyrophosphate and polyP are simply produced by heating inorganic phosphate under anhydrous conditions [7]. This is a well-described method used by manufacturers of polyphosphate glass. For example, sodium metaphosphate is manufactured by heating two parts sodium nitrate and one part phosphoric acid. Sodium metaphosphate can be prepared by the dehydration of sodium phosphate. Sodium trimetaphosphate is manufactured by heating and subsequently cooling sodium hexametaphosphate at 500°C for 8 to 12 hours [8]. In light of this, it is easy to see how polyP could be abiotically accumulated at high temperatures under anhydrous conditions during formation of the primitive Earth in which the accretion of material was heated at the core and released as steam into the atmosphere. Similar to phylosilicates, phosphoric acid salts could also bring water to the Earth's surface [9]. Additionally, it was shown that marine volcanic activity could produce water-soluble polyphosphates through partial hydrolysis of longer polyPs [3].

(ii) Second, known polyphosphate kinases (PPKs), enzymes that can mediate the synthesis and degradation of polyP chains [10], are widely distributed in microorganisms. In fact, polyP is found in each type of cell in nature [6,11].

(iii) Third, polyP can help organisms adapt to extreme conditions such as salinity, osmolarity, desiccation, UV radiation, barometric pressure, pH and temperature [12,13]. Such adaptations could have been useful for the first primitive organisms living in the conditions of a primitive Earth [14]. It was reported that *ppk1 *mutants lacking polyP are more sensitive to hydrogen peroxide, high temperatures and salt levels as compared to the wild type [15].

## PolyP in living cells

PolyP was first found as metachromatic granules in the cytoplasm of the bacterium *Spirillum volutans*, and so it was referred to as "volutin". These particles were stained pink by basic toluidine blue and were later found in other microorganisms [16]. Using electron microscopy, "volutin" granules were seen to be highly refractive and appeared to volatilize while viewed under the electron beam. Correlation between the microscopically observed number of volutin granules and the polyP cell count led to the identification of their main component as polyP. "Volutin" granules were then renamed polyP granules [17]. PolyP has since been found to be present in every cell in nature including bacterial, fungal, plant and animal cells [11].

PolyP granules contain "acid-insoluble" polyP with long-chains [2,18] and are present in the cytoplasm of various prokaryotes [6,11]. In bacterial cells, there is also "acid-soluble" polyP with short-chains [2,18] that can be found in various cell compartments (on the cell surface, in the perisplasm, and in the plasma membrane). In the *Neiseria *species, for example, polyP is capable of forming capsule-like coatings attached to the cell-surface membrane [19]. In *Helicobacter pylori*, polyP granules are detectable in the cytoplasm in association with the cell membrane; compact polyP particles can be visualized at the flagellar pole [20]. The total cellular pool of polyP depends on the phosphate concentration around the cell. Some bacteria, such as *Acinetobacter johnsonii*, accumulate up to 30% of dry cell weight [21]. PolyP granules are also known to be in eukaryotic cells, for example in trypanosomes [22], but are referred to as "acidocalcisomes." The polyP was observed as acidic, black electron-dense granules within calcium rich organelles [23]. These organelles are common in algae, plants [24], humans [25], and even in bacteria [26]. Prokaryotic cells generally lack endomembrane systems, so early suggestions that volutin granules were surrounded by a membrane [27] were ignored until H^+^-translocating pyrophosphatase, a marker for acidocalcisomes in unicellular eukaryotes, was identified by immuno-electron microscopy in the membrane surrounding polyP granules in *Agrobacterium tumefaciens *[26]. Docampo and co-workers recently reviewed acidocalcisomes. The authors presented an explanation of the presence of acidocalcisomes in both prokaryotes and eukaryotes as being of ancestral origin; this occurred before the divergence of prokaryotes and eukaryotes; and they see a convergent evolution of the polyP granules at all basic cell types to be unlikely [28].

Microscopic localisation of polyP is important for understanding its function. In the past, Kornberg and his group reviewed and proposed various alternative cell functions for polyP [6,29], showing that not only is polyP a means of storing energy [10] but it also acts as a reservoir for phosphate [30], a chelator of metal ions [31], a buffer against alkali ions [32], a channel for DNA entry [33], a regulator of stress and survival [6] and a supportive component in gene regulation [34]. In microorganisms, polyP is directly linked to physiological processes including mobility, biofilm development, quorum sensing and virulence [35,36].

Enzymes connected to the energy metabolism of polyP are polyphosphate kinase (PPK) [10], polyphosphate: glucose-6-phosphotransferase [37], exopolyphosphatase (PPX) [38], polyphosphate: adenosine monophosphate phosphotransferase (PAP) [39], 1,3- diphosphoglycerate: polyphosphate phosphotransferase [40], tripolyphosphatase [41], polyphosphate glucokinase [42] and endopolyphosphatase [43]. PPKs are key enzymes because they are capable of shifting both energy and phosphate in both directions, storage or consumption, of phosphate-energy control. PPKs are found in bacteria, archaea [44], fungi [45], yeast [46], toxoplasma [47] and algae [48,49], yet they still remain elusive in mammalian and seed plant cells [50,51]. Although PPKs were not identified in mammalian cell [50-52], it is accepted that production of polyP in these cells is linked to mitochondrial respiration, polyP is required for a normal function of respiratory chain, most importantly Complex IV [53,54]. There is a suggestion that a link exists between F_1_F_0_-ATPase regulation of polyP metabolism and mitochondrial permeability transition pore activation [50].

## PPKs as energetic enzymes

### PPK1

One of the important enzymes in biosynthesis and degradation of polyPs is polyphosphate: ADP phosphotransferase, referred to as polyphosphate kinase (PPK); it was first found in *E. coli *bacteria by Kornberg [10]. *Ec*PPK (EC 2.7.4.1) is a homotetramer that contains subunits with a molecular mass of 80 kDa [55]. The enzyme is bound to cell membranes [56] and catalyses polymerization of the terminal phosphate of ATP into a polyP chain [10]. This enzyme is referred to as polyphosphate kinase 1 (PPK1) [57]. It has been discovered that the enzyme accepts all nucleotide diphosphates (NDPs) and uses a polyP chain as a phosphate donor; it shows preference for purine nucleotides. The phosphorylation efficiency of NDP substrates is as follows: ADP > GDP > UDP > CDP [58]. *Ec*PPK1 possesses a V_max _of 3700 U/mg protein (polyP_750 _degradation and ADP phosphorylation) and a value of 0.073 for the ratio (polyP_750 _degradation, ADP phosphorylation)/(polyP synthesis, accepting polyP_15 _and ATP) [57].

### PPK2

Scientists have attempted to characterise PPK1 in other organisms, but null mutants of *Pseudomonas aeruginosa *PAO1, without detectable PPK1 activity levels, still possess as much as 20% of the wild-type polyP [59]. It has been revealed that a novel enzyme PPK2, which phosphorylates GDP to GTP by using polyP as a donor [57], is coded by the PA0141 gene [44]. It was also found that PPK2 could use GTP or ATP in the synthesis of polyP chains, differing from PPK1, which exclusively use ATP [57]. *Pa*PPK2 poses a V_max _500 000 U/mg protein (polyP_15 _degradation and GDP phosphorylation) and a value 75 for the ratio (polyP_15 _degradation, GDP phosphorylation)/(polyP synthesis, using GTP) [57].

In 2008, Nocek and colleges found that many genomes encode 2 or 3 paralogs of PPK2; most of them are 1-domain PPK2s, which are about 230 residues in length. Some genomes show the presence of a longer gene with 496-544 residues, probably produced by gene duplication, and these genes produce the 2-domain PPK2. For example, the genome of *P. aeruginosa *encodes two 1-domain PPK2s (PA0141 and PA2428) and one 2-domain PPK2 (PA3455). The authors purified some 1-domain PPK2s and some 2-domain PPK2s and found that all 1-domain PPK2s exhibited polyP-dependent ADP phosphorylation activity and generated ATP, while all 2-domain PPK2s catalysed polyP-dependent phosphorylation of AMP and produced ADP. This activity, which generates ADP, is characteristic of polyP: AMP phosphotransferase (PAP) from *Acinetobacter johnsonnii *(210AA). The authors showed that the PAP protein shares a 40% sequence homology with PA3455 and contains 2 fused PPK2 domains, indicating that it is a 2-domain PPK2 [60].

### PPK3

Using a BLAST search, we identified over 500 homologs of *P. aeruginosa *PPK2 (PA0141) with distributions from 1 to 6 homologs of PPK2 in one species [61]. We selected for research *Silicibacter pomeroyi*, including 3 homologs of PPK2 [61]. The genes were cloned into *E. coli *and, after yield-activity characterisation, the first PPK2 homolog (SPO0224) revealed properties similar to *E. coli *PPK1, the second PPK2 homolog (SPO1256) revealed properties similar to *P. aeruginosa *PPK2 and the third PPK2 homolog (SPO1727) showed a distinguishing selectivity for pyrimidine NDPs [61]. We named the SPO1727 polyphosphate kinase 3 (PPK3) [61]. PPK3 uses inorganic polyP as a donor to convert CDP to CTP [61]. PPK3 can phosphorylate NDP substrates as follows: CDP > UDP > GDP > ADP. The efficiency of polyP utilisation was found to vary among the different *Sp*PPKs. *Sp*PPK2 (SPO1256) and *Sp*PPK3 (SPO1727) utilised 100% of polyP while *Sp*PPK1 (SPO0224) utilised only 30%. These results led us to hypothesise that *S. pomeroyi *uses *Sp*PPK1 (SPO0224) for polyP synthesis and energy storage while *Sp*PPK2 (SPO1256) together with *Sp*PPK3 (SPO1727) is used for polyP utility [61].

Based on this functionality, we proposed to classify polyphosphate kinases as PPK1 (poly P synthesis), PPK2 (poly P degradation with purine phosphorylation), and PPK3 (poly P degradation with pyrimidine phosphorylation). These three classes can be doubled when 2-domain PPKs are also considered (NMP-phosphorylation). This is different from protein sequence classifications, where we recognize *E. coli *PPK1 homologs (*Ec*PPK1), *P. aeruginosa *PPK2 homologs (*Pa*PPK2) and homologs of *S. cerevisiae *vacuolar transporter chaperone's (VTC) complexes (*Sc*VTCs).

### *Ec*PPK1, *Pa*PPK2 and *Sc*VTC4p molecular structures (see Figure 2)

**Figure 2:**
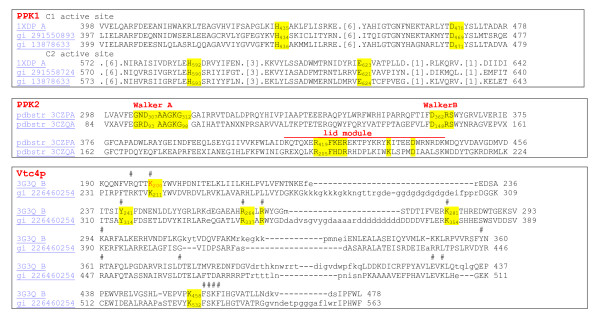
**Schematic diagram showing the key catalytic residues of PPK1, PPK2, and VTC4**.

**Figure 3:**
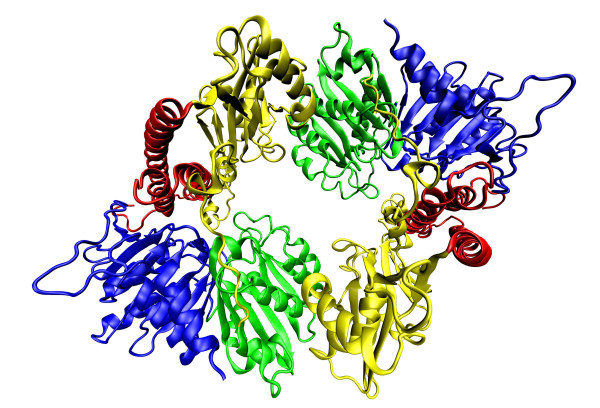
**Crystal structure of *E. coli *asymmetric unit polyphosphate kinase 1 **[62]**and its implication for polyphosphate synthesis**. PPK1 contains two asymmetric units. On the picture, there is an asymmetric unit of PPK1, which contains two monomers. Each monomer of PPK1 contains amino-terminal domain (N domain) coloured in red, the "head" domain (H domain) in yellow and two carboxyterminal domains (C1 and C2 domains) in green and blue. The coordinates were downloaded from Protein Data Bank (the corresponding PDB code- 1XDO) [63] and visualized by Visual Molecular Dynamics 1.9 [64] and POV-Ray [65].

Knowing the structures of PPKs can help us understand the functions of PPKs and also the origin and evolution of the enzymes. However, many authors have varying opinions. Some authors see similarities between *Ec*PPK1 and other polymerases such as the ribosome or RNA polymerases because everyone synthesised chains inside the tunnel [62]. They think that the *Ec*PPK1 structure may shed light on polymerase evolution as *Ec*PPK1 can be characterised as a polymerase without a template [62]. *Ec*PPK1 is also a histidine kinase because the enzyme can phosphorylate histidine residues during autophosphorylation. However, no structural similarities between *Ec*PPK1 and other histidine kinases has been found. Some structural similarities were found within the catalytic domains of phospholipase D and lipid phosphatase. The *Ec*PPK1 structure contains two asymmetric units [62] related by a pseudo two-fold symmetry that form an interlocked dimer structure; one asymmetric unit contains two monomers. Each monomer has a molecular mass of 80 kDa with 687 amino acids [55,62] and shows an L-shaped structure with four structural domains as follows: amino-terminal domain (N domain) coloured in red, the "head" domain (H domain) in yellow and two carboxyterminal domains (C1 and C2 domains) in green and blue (Figure 3) [62-65]. The N domain lies on the upper surface of the C terminal domains, consisting of 2-106 residues and forming three long antiparallel α-helixes. The H domain contains 107-321 residues and forms two α-helixes and a β-sheet between them; it forms the outward facing "head" of the monomer and interacts with the C1 domain. Both the C1 and C2 domains (residues 322-502 and 503-687) contain seven-stranded mixed β-sheet flanked by five α-helices [62].

The C1 domain is important for the first step of polyP synthesis, which involves the autophosphorylation of *Ec*PPK1 histidine residues. It was found that of the 16 histidine residues in *Ec*PPK1, 4 are conserved [62]. Mutagenesis of these 4 conserved His residues show that 2 (His-435, His-454 [62] or numbered as His-441, His-460 [66]) are important for autophosphorylation of enzymatic activity and polyP accumulation in the cell [55,66]. However, His-454 is totally buried within the hydrophobic core of the C1 domain, suggesting that His-435 is the only autophosphorylation site for *Ec*PPK1. One proposed model of autophosphorylation is that the γ-phosphate group of ATP attacks via His-435. His-592 functions as an acid, promoting the oxygen atom between the β- and γ-phosphate [62]. We recognised four conserved amino acids Glu-623, His-435, Asp-470, His-592 of the C1 and C2 domains *Ec*PPK1 that form crucial hydrogen bonds. The amino acid Glu-623 interacts with His-435 and likely plays a role in selecting the correct rotamer of His-435 by lowering the pK_a _and attacking ATP. The amino acid Asp-470 interacts with His-592 and likely facilitates in providing the correct orientation of His-592 [62]. After phosphorylation of *Ec*PPK1, the enzyme is ready to synthesise polyP chains; this process runs in a highly conserved structural tunnel, with the tunnel penetrating the centre of each *Ec*PPK1 monomer. One side of the tunnel contains a highly hydrophobic pocket that accommodates one ATP molecule, and all three phosphates are coordinated by two magnesium ions [55,62]. The other side of the tunnel contains highly conserved, positively charged residues that interact with polyP chains during elongation. It is plausible that ATP enters from one side of the tunnel and polyP chains exit from the other side [62].

**Figure 4:**
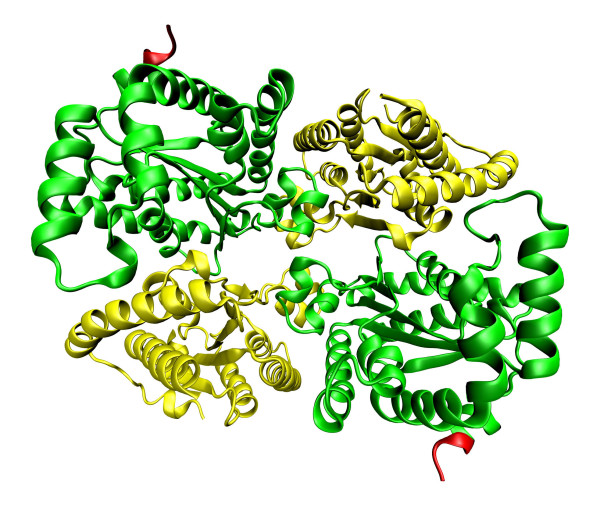
**Crystal structures of *P. aeruginosa *polyphosphate kinase 2 **[60]**and their implications for polyphosphate synthesis**. *P. aeruginosa *PPK2 contains two monomers. Each monomer contains two domains coloured in yellow and green connected by a flexible linker coloured in red. The coordinates were downloaded from Protein Data Bank (the corresponding PDB codes- 3CZP) [63] and visualized by Visual Molecular Dynamics 1.9 [64] and POV-Ray [65].

**Figure 5:**
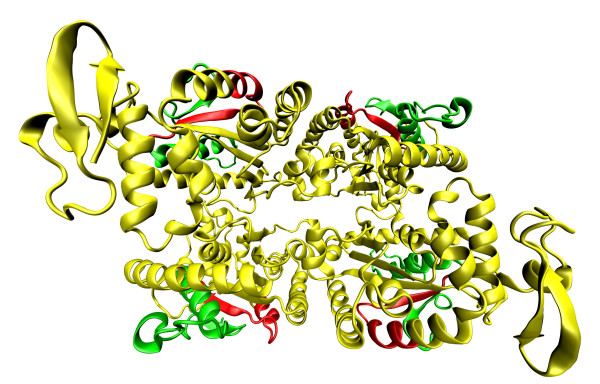
**Crystal structures of *Sinorhizobium meliloti *polyphosphate kinase 2 **[60]**and their implications for polyphosphate synthesis**. *S. meliloti *PPK2 contains four monomers. Each monomer contains two domains coloured in yellow and green connected by a flexible linker coloured in red. The coordinates were downloaded from Protein Data Bank (the corresponding PDB codes- 3CZQ) [63] and visualized by Visual Molecular Dynamics 1.9 [64] and POV-Ray [65].

*Pa*PPK2 shows structural similarities with thymidylate kinases. The conservation of key catalytic residues of thymidylate kinases in *Pa*PPK2 homologs suggests that these enzymes have a common evolutionary origin and catalytic mechanism [60]. Nocek and colleagues assembled crystal structures of PA3455 and SMc02148. They found that PA3455 is *Pa*PPK2 and has homodimeric organisation with a molecular mass of 97 kDa. Each monomer contains two PPK2 domains (residues 1-238 and 259-495) coloured in yellow and green connected by a flexible linker (residues 238-258) coloured in red (Figure 4) [60,63-65]. SMc02148 is a *Pa*PPK2 homolog from *Sinorhizobium meliloti *and contains four PPK2 monomers in an asymmetric unit with a molecular mass of 124.5 kDa (Figure 5) [60,63-65]. PA3455 and SMc02148 monomers have similar structures, with both containing N- and C- terminal PPK2 domains. Each domain contains a 3-layer α/β/α sandwich and 5 (PA3455) or 6 (SMc02148) parallel β sheets in the central location of the domain. The central β-sheet is flanked by 3 longer sheets on one side, 5 shorter sheets on the other side and 2 α-helixes at the top of the C terminal for the PA3455 domain; for the SMc02148 domain, similar structure is seen. It should be noted that the N-terminal domain was only partially modelled. The authors suggest that the active side of the enzyme is under the lid module near the 2 Walker loops (Figure 2). Walker motifs contain the conserved residues Ala-309, Gly-310, Lys-311, Gly-312, Asp-362 and Arg-423 in PA3455 and Gly-96, Lys-97, Arg-209 and Lys-218 in SMc02148. The Walker A motif binds the β- and γ-phosphates of ATP and the Asp of the Walker B motif coordinates Mg^2+ ^cations [60,67].

**Figure 6:**
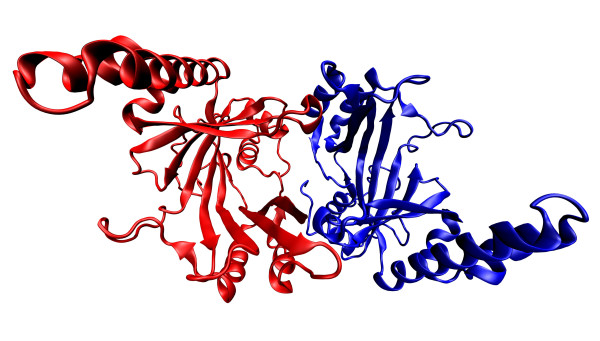
**Crystal structure of *Saccharomyces cerevisiae *VTC4 **[46]**and its implication for polyphosphate synthesis**. VTC4 contains two monomers coloured in red and blue. The coordinates were downloaded from Protein Data Bank (the corresponding PDB code- 3G3Q) [63] and visualized by Visual Molecular Dynamics 1.9 [64] and POV-Ray [65].

In *Saccharomyces cerevisiae*, polyP is accumulated in both the extracellular space and the vacuoles [68]. PolyP synthesis is connected with the vacuolar transporter chaperone complex (VTC). VTC proteins form a membrane assembly made of hetero-oligomeric proteins [69]. It is possible to distinguish the small transmembrane protein VTC1 from the three proteins that contain transmembrane domains and a cytoplasmic segment, VTC2, VTC3 and VTC4 [70]. The most interesting protein is VTC4, which is essential for accumulation of polyP in the cell. VTC4 contains two monomers coloured in blue and red (Figure 6) [46,63-65]. The structure of VTC4 contains the tunnel that generates polyP from ATP. The entire tunnel domain contains substrate-binding and acceptor pockets. It is likely that the cleaved γ-phosphate from ATP is attached by Lys-200 and then transferred into the acceptor pocket [46].

## PolyP and PPK as active metabolic regulators (see Figure 7)

**Figure 7:**
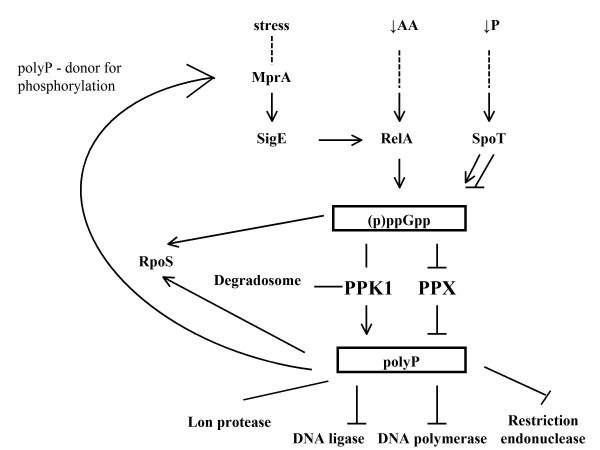
**The cenral role of PPK1 in metabolism involved in gene and protein regulation**. ↓AA - amino acid starvation; ↓P - phosphate starvation; (p)ppGpp -guanosine (penta)tetraphosphate; → activation; ⊥ inhibition.

In *E. coli*, a model prokaryote, the Pho regulon senses low concentrations of orthophosphate in the medium. Phosphate starvation in the cell is detected by PhoR, which leads to activation of the principal phosphate regulator PhoB [71]. This regulator, in turn, activates more than 30 genes, including PhoA that encodes for alkaline phosphatase and SpoT; this in turn accumulates or degrades ppGpp [72]. Amino acid starvation in *E. coli *leads to the activation of RelA, which is responsible for a massive accumulation of guanosine (penta) tetraphosphate (p)ppGpp [73]. It has been reported that low phosphate concentrations and low concentrations of amino acids in the growth medium are required for polyP accumulation. Thus, a mutant lacking both genes, RelA and SpoT, accumulates neither (p)ppGpp nor polyP [74]. PpGpp controls bacterial transcription, translation and replication [75], so the connection between (p)ppGpp and polyP indicates a broader role for polyP in cell regulation. For example, it has been reported that polyP plays a central role in the stress response of mycobacterium, where PPK1 is required for the MprAB-sigE-rel signalling system [76]. The presence of PPK1 leads to transcription of the two-signal transduction system MprAB, which in turn regulates the expression of SigE, a stress regulated δ^E^-factor. δ^E ^regulates transcription of the ppGpp regulator RelA [76]. It seems that under stress conditions, polyP is a preferred donor for phosphorylation of MprA [77], a cytoplasmic response regulator. MprA then binds the promoter of the MprAB operon to initiate transcription, providing a positive feedback loop in which production of MprA brings further MprA synthesis. In this way, the MprAB operon reaches a basal level of gene expression [77,78]. If the MprAB operon is activated, phosphorylated MprA increases transcription of the gene SigE and consecutively increases transcription of Rel-ppGpp synthesis in *Mycobacterium smegmatis *and *M. tuberculosis *[76].

It has been reported that the activities of enzymes that both synthesise and degrade polyP chains fluctuate only marginally [74]. For these reasons, turnover of polyP is generated by cyclic hydrolytic breakdown by exopolyphosphatase (PPX) and synthetic accumulation by PPK [58,74]. PPX is an enzyme that degrades polyP and releases orthophosphate from the ends of polyP [38]. It has been demonstrated that pppGpp inhibits *E. coli *PPX but not *Ec*PPK, which in turn leads to a 100- to 1000-fold accumulation of polyP [58]. The coordinated regulation of *Ec*PPX and *Ec*PPK activities is not surprising. The *E. coli *genes for PPX are located downstream of Ppk and are organized in a co-linear arrangement, thus forming an operon. This means that the level of polyP degradation activity by *Ec*PPX is always dependent on the polyP synthesis level of *Ec*PPK [56]. Another example can be found in *P. aeruginosa*. The PPX homologue gene in *P. aeruginosa *is located in a direction opposite of Ppk1, and so they do not form an operon. This difference may account for the difference in polyP levels among different bacteria [79]. PolyP accumulation in *P. aeruginosa *is several-fold greater than in *E. coli *[80]. It appears that *Pa*PPX enzyme levels are regulated independently of the *Pa*PPK1 levels [79]. A microarray analysis indicated that PPK1 has a central role in gene regulation. The DNA microarrays showed changes in mRNA levels of the *P. aeruginosa ppk1 *mutant; it was observed that 240 genes were up-regulated and 460 genes were down-regulated. In the case of the *P. aeruginosa ppk2 *mutant, only 20 genes were up- or down-regulated [81]. Overexpression of the *E. coli *Ppk1 gene increased polyphosphate: AMP phosphotransferase (PAP) activity drastically. Investigation of this mechanism revealed that *Ec*PPK1 overproduction enhanced the activity of adenylate kinase and expressed PAP activity [82]. PPK1 has important regulation roles in microbial cells and it is not found in higher eukaryotes. Thus, PPK1 has been suggested as a potential target for antibiotics [83].

### PolyP, PPK and mRNA connections

As mentioned above, cell starvation of phosphate, nitrogen, amino acids and other nutrients induces a stress response signal that generates (p)ppGpp [73,84]. These nucleotides repress many genes, including those for ribosome synthesis, and activate 50 or more genes responsible for coping with stress and starvation [85]. Accumulation of (p)ppGpp in *E. coli *plays a major regulatory role in synthesis of the stationary-phase specific RNA polymerase sigma factor (δ^S^), which is encoded by the RpoS gene [15,86] and leads to initiation of the stationary phase [73,84]. It was reported that polyP is also necessary for induction of the transcription factor RpoS [86]. δ^S ^is a major player in the regulation of gene expression in the stationary phase, and is activated in response to various stresses including nutrient limitations and osmotic challenges [87]; more than 30 genes show *RpoS*-dependent expression in *E. coli *[88].

PPK1 is a component of the *E. coli *degradosome and plays a role in mRNA degradation. *Ec*PPK1 does not bind to RNA at the 3' or 5' terminal phosphate, but has to bind along the backbone; RNA binding activity involves the active centre of the enzyme. *Ec*PPK1 may promote assembly of the degradosome, or its interaction with the RNA may maintain an appropriate microenvironment by removing inhibitory polyphosphates. PolyP is a potential inhibitor of mRNA degradation by the degradosome [89]. It was reported that, in vitro, polyP inhibits other nucleic acid-modifying enzymes such as DNA ligase, restriction endonuclease and DNA polymerase [90]. PPK can bind and degrade inhibitory polyP in the presence of ADP [89] or it can participate in the cyclic hydrolytic breakdown of polyP by PPX [58,74]. As PPK is inhibited, the mRNA half-life in vivo is decreased [90]. As PPK degrades polyP, ADP is removed. ADP is a potential inhibitor of polynucleotide phosphorylase [91] in the degradosome. Regeneration of ATP by PPK is required for RhlB helicase activity [89].

### PolyP, PPK and protein connections

In *E. coli*, the degradation of most cytoplasmic proteins consumes ATP [92]; ATP-dependent protease Lon is mainly involved in this process [93,94]. Kuroda and colleagues found that during stress, the Lon protease forms a complex with polyP. The polyP-Lon complex is very large because one molecule of polyP binds to four molecules of Lon. This complex degrades free ribosomal proteins [95]. The degradation of intracellular proteins can be important in cell responses to stress; this generates free amino acids that can be used as an immediately accessible source needed in the synthesis of new stress-response proteins, such as regulatory enzymes and transporters [96,97].

PolyP and DNA compete to bind Lon. The binding sites are localised in the same ATPase domain of Lon protease, and it seems that Lon has a higher affinity for polyP than for DNA [98]. Some studies show that Lon controls the level of mRNA transcription for the *E. coli *gal operon [99]. *E. coli *Lon proteases look like DNA-binding proteins but with low specificities. The drastic change in intracellular soluble polyP levels can affect the DNA-binding ability of Lon and its regulation of cellular functions [100,101]. It was shown that polyP stimulates translation in vitro [102]. McInerney and colleges showed that polyP could also interact with intact ribosomes, where the strongest points of attachment were on the protein components of the ribosome. PolyP attaches to both the 50S and 30S subunits of ribosomes [103].

Group II introns are ribozymes as well as bacterial mobile elements thought to be ancestors of both introns (genetic material that is discarded from messenger RNA transcripts) and retroelements (genetic elements and viruses that replicate via reverse transcription) in all three domains of life. *Lactococcus lactis *catalytically activates intron RNA (Ll.LtrB) and an intron-encoded reverse transcriptase (LtrA) from ribonucleoprotein particles localized in the cellular poles of bacteria. Zhao J. and co-workers used fluorescence microscopy with cell microarrays to screen a transposon-insertion library for mutants with altered LtrA localisations. They found that LtrA localisation in the mutants was affected by the accumulation of intracellular polyP. PolyP delocalized ribonucleoprotein particles away from the cellular poles. Thus, polyP serves as a potential regulator of protein localisation with wide physiological consequences [104].

## Possible PPK applications in industry

As described above, Arthur Kornberg (Nobel Prize in Physiology or Medicine, 1959), together with his wife Sylvy Ruth and Simms E. S., identified PPK for the first time in *E. coli *in 1956 [10]. In the following year, Kornberg S.R. showed the reverse reaction and proposed it was a system for ATP synthesis [105]. After 20 years, the ATP-regeneration system based on polyP and *Ec*PPK1 has been suggested for use in enzyme technology applications [106]. In this system, a reaction mixture, with ADP and polyP, is percolated through a column containing immobilized *Ec*PPK1. The ATP-enriched mixture can then be used in the next reaction [106]. However, isolation of the cell extract while maintaining high *Ec*PPK1 activities proved to be difficult; because *E. coli *cells are rich in ATP-degrading enzymes, a simple separation process from ATP-hydrolysing activities was still needed [107]. Hoffman and co-workers (1988) purified *E. coli *lysate enough to stop the ATP hydrolysis activity through ammonium sulphate precipitation and DEAE cellulose fractionation. They obtained 390 mg of *Ec*PPK1 from 1 kg of fresh cell paste and immobilised the enzyme using glutaraldehyde-activated (2-aminoethyl) cellulose, which decreased the enzymatic activity to 10.6% [107]. Production and immobilisation of the enzyme was later improved by recombinant DNA technology. His-tagged *Ec*PPK1 was easily produced and immobilised on a nickel chelating resin, yet the ATP-regeneration process was unfortunately unstable [108]. It was found that overproduction of *Ec*PPK1 in *E. coli *leads to accumulation of inclusion bodies, and that the inclusion bodies are sufficiently pure and surprisingly active [109]. When these inclusion bodies were entrapped in agar/TiO_2 _beads the ATP-regeneration process was stable, and the system was again suggested for use in enzyme technology applications [109]. The basic disadvantage of the proposed system has been the low "total turnover number" (TTN), which is the total moles of product formed per mole of cofactor during the course of a complete reaction [110]. In light of this, other ATP-regeneration systems, such as acetyl phosphate and acetate kinase [111], phosphoenol pyruvate and pyruvate kinase [112] and creatine phosphate and creatine kinase [113] proved to be more attractive for enzyme technology applications. For example, GeneChem, Inc. uses an acetyl phosphate and acetate kinase regeneration system for the production process of CMP-NeuAc and sialyllactose [114]. Recently, our group successfully used *S. pomeroyi *PPK3 in the same process at a laboratory scale. The characteristics of *Sp*PPK3, such as high TNT, easy immobilisation and easy separation from NTP hydrolysing activities, will hopefully lead the way to a broader spectrum of enzyme technology applications [61].

Some technological applications using thermophilic enzymes require a higher temperature resistant ATP-regeneration system. PPK from *Thermus thermophilus*, which shows a 30% amino acid sequence homology to *Ec*PPK1, generated fructose 1,6-diphosphate for at least one week at 70°C [115]. Sato and colleges studied ATP-requiring D-amino acid dipeptide synthesis using PPK from *Thermosynechococcus *[116], but this enzyme was less thermostable than *Thermus thermophilus *PPK [115].

## Applications in synthetic biology

**Figure 8:**
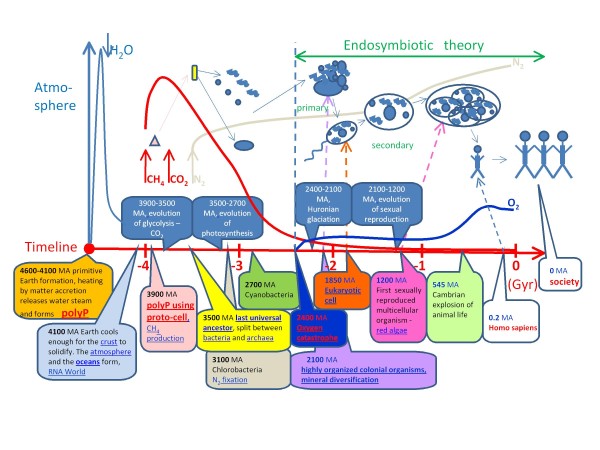
**Timeline of key events in the global history of life evolution, integrating evidence for the endosymbiotic theory and insertion of the hypothesis of polyP-protocells**. This scheme was designed to depict synthetic biology section. MA - million years ago.

"Synthetic biology" is a scientific area that includes two intentions. One area uses unnatural molecules to reproduce emergent behaviours in natural biology with the goal of creating artificial life. The other area seeks interchangeable parts from natural biology to assemble systems that function unnaturally [117]. In both cases, the intentions are focused on a better understanding of life and on the use of knowledge for a commercial benefit. For example, the design and construction of minimal cells, one main goal of synthetic biology [118], would be beneficial for the biotechnology industry. Steps towards this have already been performed; a chemically synthesised genome was successfully transplanted into *M. capricolum *bacterial cells [119]. Designing and programming synthetic life forms based on new DNA software, which includes the use of new cell materials, components and metabolic schemes, is a process coming in the near future. In terms of simplicity of the minimal genome, polyP represents an ideal energy source to power all vital functions within a synthetic cell. It could completely replace photosynthesis, respiration, glycolysis and other alternative energy sources within the cell. As mentioned above, polyphosphate is present in all natural life forms, but none use it as an essential energy source. To explain this phenomenon, this review examined some logical references that led to a hypothesis that polyP is a molecule connected to life creation. PolyP could have been abiotically accumulated at high temperatures and under anhydrous conditions during formation of the primitive Earth, when the accretion of material heated the core and released steam to the atmosphere. The presence of acidocalcisomes in both prokaryotes and eukaryotes represents the ancestral origin before the divergence of prokaryotes and eukaryotes. Moreover, the membrane surrounding acidocalcisomes inside prokaryotic cells could have endosymbiotic origins (Figure 8). The endosymbiotic hypothesis is almost as old as Darwin's theory. Some botanists observed structural similarities between chloroplasts and *Cyanobacteria *at the end of 19^th ^century. However, only Lynn Margulis-Sagan shifted it to theory in 1967 [120], and only recently has supportive evidence been found. The theory argues that eukaryotic cells originated from highly organized colonial organisms in connection with an "oxygen catastrophe" and "huronian glaciations" (Figure 8) 2400-2100 million years ago (MA). The prokaryotes and archaea had to react to the atmospheric poisoning by oxygen and to the age of the "snowball Earth". This supports the adaptationist model of evolution [121] where the model inevitably leads to the concept of "progress" (i.e., gradual improvement of "organs"). In evolution, successful events are conserved and integrated into developmental mechanisms, so it is no surprise that the establishment of the eukaryotic cell led to secondary endosymbiosis; this provided massive gene transfer between eukaryotes [122] and, eventually, the process led to sexual reproduction and multicellular organisms (Figure 8). The multicellular organism represents a society of highly differentiated cells with sophisticated intercommunication languages. Development of human society with languages is nothing new; the evolutionary processes remain, essentially, the same throughout the history of life. For example, even the social collective behaviour could be integrated into the evolutionary processes much earlier on the bacterial [123], viral, or gene levels. Considering this, the integration of endosymbiotic principles into the evolutionary process could be extrapolated deep into the past. We propose a model where polyP granules, or acidocalcisomes, have an ancestral endosymbiotic origin (Figure 8). If this is true, it will strongly support the sense of construction of proto-cells which use polyP as an ancient source of energy. Only two genes are needed for polyP utilisation, namely PPK and PPX. This is very interesting for design of minimal cells, a main goal of synthetic biology.

## Conclusion

Inorganic polyP was probably present on Earth at the prebiotic time. At present, it is a molecule of many functions involved in energetic metabolism and gene regulation. These two processes are orchestrates by PPK enzymes that can mediate the synthesis and degradation of polyP chain.

This review prepared some references for the response why polyphosphate is present in all natural life forms. Conclusion leads to a hypothesis that polyP is a molecule connected to life creation. PolyP could have been abiotically accumulated at high temperatures and under anhydrous conditions during formation of primitive Earth; the catalytic core inside the tunnel structure of PPK could be characterized as a RNA polymerase without the template; polyP and PPKs are connected with RNA and protein regulation; and the membrane surrounding of acidocalcisomes inside the prokaryotic cell could imply endosymbiotic origin. These indications could be used in synthetic biology and microbial technologies for a minimisation of the genomic software. Besides, *Sp*PPK3 has already demonstrated to be well NTP-regeneration system (cheap substrate and high TTN - total moles of product formed per mole of cofactor) that hopefully will initiate a broader spectrum of enzyme technology applications.

## Competing interests

The authors declare that they have no competing interests.

## Authors' contributions

All authors read and approved the final manuscript.

## References

[B1] Lodish H Molecular cell biology 2007 6 New York: W.H. Freeman

[B2] Kulaev IS Biochemistry of inorganic polyphosphates Rev Physiol Biochem Pharmacol 1975 73 131 158 10.1007/BFb0034661 175427

[B3] Yamagata Y Watanabe H Saitoh M Namba T Volcanic production of polyphosphates and its relevance to prebiotic evolution Nature 1991 352 516 519 10.1038/352516a0 11536483

[B4] Gower LB Biomimetic model systems for investigating the amorphous precursor pathway and its role in biomineralization Chem Rev 2008 108 4551 4627 10.1021/cr800443h 19006398 PMC3652400

[B5] Diaz J Ingall E Benitez-Nelson C Paterson D de Jonge MD McNulty I Brandes JA Marine polyphosphate: a key player in geologic phosphorus sequestration Science 2008 320 652 655 10.1126/science.1151751 18451299

[B6] Kornberg A Inorganic polyphosphate: toward making a forgotten polymer unforgettable J Bacteriol 1995 177 491 496 7836277 10.1128/jb.177.3.491-496.1995 PMC176618

[B7] Baltscheffsky H Blomberg C Liljenstrom H Lindahl BI Arhem P On the origin and evolution of life: an introduction J Theor Biol 1997 187 453 459 10.1006/jtbi.1996.0380 9299290

[B8] Budavari S The Merck index: An encyclopedia of chemicals, drugs, and biologicals 1989 11 Rahway, NJ: Merck & Co

[B9] Ciesla F Lauretta D Radial migration and dehydration of phyllosilicates in the solar nebula Earth and Planetary Science Letters 2005 231 1 8 10.1016/j.epsl.2004.12.022

[B10] Kornberg A Kornberg SR Simms ES Metaphosphate synthesis by an enzyme from Escherichia coli Biochim Biophys Acta 1956 20 215 227 13315368 10.1016/0006-3002(56)90280-3

[B11] Kulaev IS Vagabov VM Polyphosphate metabolism in micro-organisms Adv Microb Physiol 1983 24 83 171 6320606 10.1016/s0065-2911(08)60385-9

[B12] Rothschild LJ Mancinelli RL Life in extreme environments Nature 2001 409 1092 1101 10.1038/35059215 11234023

[B13] Seufferheld MJ Alvarez HM Farias ME Role of polyphosphates in microbial adaptation to extreme environments Appl Environ Microbiol 2008 74 5867 5874 10.1128/AEM.00501-08 18708516 PMC2565944

[B14] Stribling R Miller SL Energy yields for hydrogen cyanide and formaldehyde syntheses: the HCN and amino acid concentrations in the primitive ocean Orig Life Evol Biosph 1987 17 261 273 10.1007/BF02386466 2819806

[B15] Rao NN Kornberg A Inorganic polyphosphate supports resistance and survival of stationary-phase Escherichia coli J Bacteriol 1996 178 1394 1400 8631717 10.1128/jb.178.5.1394-1400.1996 PMC177814

[B16] Meyer A Orientierende Untersuchungen ueber Verbreitung, Morphologie, und Chemie des Volutins Bot Zeit 1904 62 113 152

[B17] Wiame JM The metachromatic reaction of hexametaphosphate J Am Chem Soc 1947 69 3146 18919721 10.1021/ja01204a508

[B18] Wood HG Clark JE Biological aspects of inorganic polyphosphates Annu Rev Biochem 1988 57 235 260 10.1146/annurev.bi.57.070188.001315 3052272

[B19] Tinsley CR Manjula BN Gotschlich EC Purification and characterization of polyphosphate kinase from Neisseria meningitidis Infect Immun 1993 61 3703 3710 8395468 10.1128/iai.61.9.3703-3710.1993 PMC281067

[B20] Bode G Mauch F Ditschuneit H Malfertheiner P Identification of structures containing polyphosphate in Helicobacter pylori J Gen Microbiol 1993 139 3029 3033 8126429 10.1099/00221287-139-12-3029

[B21] Deinema MH van Loosdrecht M Scholten A Some physiological characteristics of Acinetobacter spp. accumulating large amounts of phosphate Wat Sci Technol 1985 17 119 125

[B22] Swellengrebel NH La volutine chez les trypanosomes C R Soc Biol 1908 64 38 43

[B23] LeFurgey A Ingram P Blum JJ Elemental composition of polyphosphate-containing vacuoles and cytoplasm of Leishmania major Mol Biochem Parasitol 1990 40 77 86 10.1016/0166-6851(90)90081-V 2348832

[B24] Ruiz FA Marchesini N Seufferheld M Docampo R The polyphosphate bodies of Chlamydomonas reinhardtii possess a proton-pumping pyrophosphatase and are similar to acidocalcisomes J Biol Chem 2001 276 46196 46203 10.1074/jbc.M105268200 11579086

[B25] Ruiz FA Lea CR Oldfield E Docampo R Human platelet dense granules contain polyphosphate and are similar to acidocalcisomes of bacteria and unicellular eukaryotes J Biol Chem 2004 279 44250 44257 10.1074/jbc.M406261200 15308650

[B26] Seufferheld M Vieira MC Ruiz FA Rodrigues CO Moreno SN Docampo R Identification of organelles in bacteria similar to acidocalcisomes of unicellular eukaryotes J Biol Chem 2003 278 29971 29978 10.1074/jbc.M304548200 12783865

[B27] Friedberg I Avigad G Structures containing polyphosphate in Micrococcus lysodeikticus J Bacteriol 1968 96 544 553 5674060 10.1128/jb.96.2.544-553.1968 PMC252328

[B28] Docampo R Ulrich P Moreno SN Evolution of acidocalcisomes and their role in polyphosphate storage and osmoregulation in eukaryotic microbes Philos Trans R Soc Lond B Biol Sci 2010 365 775 784 10.1098/rstb.2009.0179 20124344 PMC2817225

[B29] Kornberg A Inorganic polyphosphate: a molecule of many functions Prog Mol Subcell Biol 1999 23 1 18 10.1007/978-3-642-58444-2_1 10448669

[B30] Harold FM Inorganic polyphosphates in biology: structure, metabolism, and function Bacteriol Rev 1966 30 772 794 5342521 10.1128/br.30.4.772-794.1966 PMC441015

[B31] Archibald FS Fridovich I Investigations of the state of the manganese in Lactobacillus plantarum Arch Biochem Biophys 1982 215 589 596 10.1016/0003-9861(82)90120-5 6284057

[B32] Pick U Weiss M Polyphosphate Hydrolysis within Acidic Vacuoles in Response to Amine-Induced Alkaline Stress in the Halotolerant Alga Dunaliella salina Plant Physiol 1991 97 1234 1240 10.1104/pp.97.3.1234 16668514 PMC1081147

[B33] Castuma CE Huang R Kornberg A Reusch RN Inorganic polyphosphates in the acquisition of competence in Escherichia coli J Biol Chem 1995 270 12980 12983 10.1074/jbc.270.22.12980 7768888

[B34] Tsutsumi K Munekata M Shiba T Involvement of inorganic polyphosphate in expression of SOS genes Biochim Biophys Acta 2000 1493 73 81 10978509 10.1016/s0167-4781(00)00165-2

[B35] Rashid MH Kornberg A Inorganic polyphosphate is needed for swimming, swarming, and twitching motilities of Pseudomonas aeruginosa Proc Natl Acad Sci USA 2000 97 4885 4890 10.1073/pnas.060030097 10758151 PMC18327

[B36] Rashid MH Rao NN Kornberg A Inorganic polyphosphate is required for motility of bacterial pathogens J Bacteriol 2000 182 225 227 10.1128/JB.182.1.225-227.2000 10613886 PMC94263

[B37] Szymona M Ostrowski W Inorganic Polyphosphate Glucokinase of Mycobacterium Phlei Biochim Biophys Acta 1964 85 283 295 14212975 10.1016/0926-6569(64)90249-4

[B38] Akiyama M Crooke E Kornberg A An exopolyphosphatase of Escherichia coli. The enzyme and its ppx gene in a polyphosphate operon J Biol Chem 1993 268 633 639 8380170

[B39] Dirheimer G Ebel JP Characterization of a Polyphosphate-Amp-Phosphotransferase in Corynebacterium Serosis C R Hebd Seances Acad Sci 1965 260 3787 3790 14339663

[B40] Kulaev IS Biochemistry of inorganic polyphosphates 1979 J Wiley and Sons, Chichester

[B41] van Alebeek GJ Keltjens JT van der Drift C Purification and characterization of inorganic pyrophosphatase from Methanobacterium thermoautotrophicum (strain delta H) Biochim Biophys Acta 1994 1206 231 239 10.1016/0167-4838(94)90213-5 8003527

[B42] Hsieh PC Shenoy BC Jentoft JE Phillips NF Purification of polyphosphate and ATP glucose phosphotransferase from Mycobacterium tuberculosis H37Ra: evidence that poly(P) and ATP glucokinase activities are catalyzed by the same enzyme Protein Expr Purif 1993 4 76 84 10.1006/prep.1993.1012 8381043

[B43] Lichko LP Kulakovskaya TV Kulaev IS Properties of partially purified endopolyphosphatase of the yeast Saccharomyces cerevisiae Biochemistry (Mosc) 2010 75 1404 1407 10.1134/S0006297910110131 21314609

[B44] Zhang H Ishige K Kornberg A A polyphosphate kinase (PPK2) widely conserved in bacteria Proc Natl Acad Sci USA 2002 99 16678 16683 10.1073/pnas.262655199 12486232 PMC139203

[B45] Tani C Ohtomo R Osaki M Kuga Y Ezawa T ATP-dependent but proton gradient-independent polyphosphate-synthesizing activity in extraradical hyphae of an arbuscular mycorrhizal fungus Appl Environ Microbiol 2009 75 7044 7050 10.1128/AEM.01519-09 19767467 PMC2786526

[B46] Hothorn M Neumann H Lenherr ED Wehner M Rybin V Hassa PO Uttenweiler A Reinhardt M Schmidt A Seiler J Ladurner AG Herrmann C Scheffzek K Mayer A Catalytic core of a membrane-associated eukaryotic polyphosphate polymerase Science 2009 324 513 516 10.1126/science.1168120 19390046

[B47] Rooney PJ Ayong L Tobin CM Moreno SN Knoll LJ TgVTC2 is involved in polyphosphate accumulation in Toxoplasma gondii Mol Biochem Parasitol 2011 176 121 126 10.1016/j.molbiopara.2010.12.012 21195114 PMC3042031

[B48] Gomez-Garcia MR Kornberg A Formation of an actin-like filament concurrent with the enzymatic synthesis of inorganic polyphosphate Proc Natl Acad Sci USA 2004 101 15876 15880 10.1073/pnas.0406923101 15496465 PMC528760

[B49] Yagisawa F Nishida K Yoshida M Ohnuma M Shimada T Fujiwara T Yoshida Y Misumi O Kuroiwa H Kuroiwa T Identification of novel proteins in isolated polyphosphate vacuoles in the primitive red alga Cyanidioschyzon merolae Plant J 2009 60 882 893 10.1111/j.1365-313X.2009.04008.x 19709388

[B50] Pavlov E Aschar-Sobbi R Campanella M Turner RJ Gomez-Garcia MR Abramov AY Inorganic polyphosphate and energy metabolism in mammalian cells J Biol Chem 2010 285 9420 9428 10.1074/jbc.M109.013011 20124409 PMC2843191

[B51] Hooley P Whitehead MP Brown MR Eukaryote polyphosphate kinases: is the 'Kornberg' complex ubiquitous? Trends Biochem Sci 2008 33 577 582 10.1016/j.tibs.2008.09.007 18938082

[B52] Kumble KD Kornberg A Inorganic polyphosphate in mammalian cells and tissues J Biol Chem 1995 270 5818 5822 10.1074/jbc.270.11.5818 7890711

[B53] Pavlov E Zakharian E Bladen C Diao CT Grimbly C Reusch RN French RJ A large, voltage-dependent channel, isolated from mitochondria by water-free chloroform extraction Biophys J 2005 88 2614 2625 10.1529/biophysj.104.057281 15695627 PMC1305358

[B54] Abramov AY Fraley C Diao CT Winkfein R Colicos MA Duchen MR French RJ Pavlov E Targeted polyphosphatase expression alters mitochondrial metabolism and inhibits calcium-dependent cell death Proc Natl Acad Sci USA 2007 104 18091 18096 10.1073/pnas.0708959104 17986607 PMC2084301

[B55] Ahn K Kornberg A Polyphosphate kinase from Escherichia coli. Purification and demonstration of a phosphoenzyme intermediate J Biol Chem 1990 265 11734 11739 2164013

[B56] Akiyama M Crooke E Kornberg A The polyphosphate kinase gene of Escherichia coli. Isolation and sequence of the ppk gene and membrane location of the protein J Biol Chem 1992 267 22556 22561 1331061

[B57] Ishige K Zhang H Kornberg A Polyphosphate kinase (PPK2), a potent, polyphosphate-driven generator of GTP Proc Natl Acad Sci USA 2002 99 16684 16688 10.1073/pnas.262655299 12482933 PMC139204

[B58] Kuroda A Kornberg A Polyphosphate kinase as a nucleoside diphosphate kinase in Escherichia coli and Pseudomonas aeruginosa Proc Natl Acad Sci USA 1997 94 439 442 10.1073/pnas.94.2.439 9012801 PMC19530

[B59] Rashid MH Rumbaugh K Passador L Davies DG Hamood AN Iglewski BH Kornberg A Polyphosphate kinase is essential for biofilm development, quorum sensing, and virulence of Pseudomonas aeruginosa Proc Natl Acad Sci USA 2000 97 9636 9641 10931957 10.1073/pnas.170283397 PMC16917

[B60] Nocek B Kochinyan S Proudfoot M Brown G Evdokimova E Osipiuk J Edwards AM Savchenko A Joachimiak A Yakunin AF Polyphosphate-dependent synthesis of ATP and ADP by the family-2 polyphosphate kinases in bacteria Proc Natl Acad Sci USA 2008 105 17730 17735 10.1073/pnas.0807563105 19001261 PMC2584756

[B61] Nahalka J Patoprsty V Enzymatic synthesis of sialylation substrates powered by a novel polyphosphate kinase (PPK3) Org Biomol Chem 2009 7 1778 1780 10.1039/b822549b 19590770

[B62] Zhu Y Huang W Lee SS Xu W Crystal structure of a polyphosphate kinase and its implications for polyphosphate synthesis EMBO Rep 2005 6 681 687 10.1038/sj.embor.7400448 15947782 PMC1369109

[B63] Berman HM Westbrook J Feng Z Gilliland G Bhat TN Weissig H Shindyalov IN Bourne PE Protein Data Bank Nucleic Acids Research 2000 28 235 242 10.1093/nar/28.1.235 10592235 PMC102472

[B64] Humphrey W Dalke A Schulten K Visual Molecular Dynamics Journal of Molecular Graphics 1996 14 33 38 10.1016/0263-7855(96)00018-5 8744570

[B65] POV-Ray version 3.6.2 http://www.povray.org/

[B66] Kumble KD Ahn K Kornberg A Phosphohistidyl active sites in polyphosphate kinase of Escherichia coli Proc Natl Acad Sci USA 1996 93 14391 14395 10.1073/pnas.93.25.14391 8962061 PMC26142

[B67] Leipe DD Koonin EV Aravind L Evolution and classification of P-loop kinases and related proteins J Mol Biol 2003 333 781 815 10.1016/j.jmb.2003.08.040 14568537

[B68] Werner TP Amrhein N Freimoser FM Specific localization of inorganic polyphosphate (poly P) in fungal cell walls by selective extraction and immunohistochemistry Fungal Genet Biol 2007 44 845 852 10.1016/j.fgb.2007.01.008 17320430

[B69] Ogawa N DeRisi J Brown PO New components of a system for phosphate accumulation and polyphosphate metabolism in Saccharomyces cerevisiae revealed by genomic expression analysis Mol Biol Cell 2000 11 4309 4321 11102525 10.1091/mbc.11.12.4309 PMC15074

[B70] Muller O Neumann H Bayer MJ Mayer A Role of the Vtc proteins in V-ATPase stability and membrane trafficking J Cell Sci 2003 116 1107 1115 10.1242/jcs.00328 12584253

[B71] Makino K Shinagawa H Amemura M Kawamoto T Yamada M Nakata A Signal transduction in the phosphate regulon of Escherichia coli involves phosphotransfer between PhoR and PhoB proteins J Mol Biol 1989 210 551 559 10.1016/0022-2836(89)90131-9 2693738

[B72] Gentry DR Cashel M Mutational analysis of the Escherichia coli spoT gene identifies distinct but overlapping regions involved in ppGpp synthesis and degradation Mol Microbiol 1996 19 1373 1384 10.1111/j.1365-2958.1996.tb02480.x 8730877

[B73] Gentry DR Hernandez VJ Nguyen LH Jensen DB Cashel M Synthesis of the stationary-phase sigma factor sigma s is positively regulated by ppGpp J Bacteriol 1993 175 7982 7989 8253685 10.1128/jb.175.24.7982-7989.1993 PMC206978

[B74] Rao NN Liu S Kornberg A Inorganic polyphosphate in Escherichia coli: the phosphate regulon and the stringent response J Bacteriol 1998 180 2186 2193 9555903 10.1128/jb.180.8.2186-2193.1998 PMC107147

[B75] Srivatsan A Wang JD Control of bacterial transcription, translation and replication by (p)ppGpp Curr Opin Microbiol 2008 11 100 105 10.1016/j.mib.2008.02.001 18359660

[B76] Sureka K Dey S Datta P Singh AK Dasgupta A Rodrigue S Basu J Kundu M Polyphosphate kinase is involved in stress-induced mprAB-sigE-rel signalling in mycobacteria Mol Microbiol 2007 65 261 276 10.1111/j.1365-2958.2007.05814.x 17630969

[B77] Zahrt TC Wozniak C Jones D Trevett A Functional analysis of the Mycobacterium tuberculosis MprAB two-component signal transduction system Infect Immun 2003 71 6962 6970 10.1128/IAI.71.12.6962-6970.2003 14638785 PMC308901

[B78] He H Zahrt TC Identification and characterization of a regulatory sequence recognized by Mycobacterium tuberculosis persistence regulator MprA J Bacteriol 2005 187 202 212 10.1128/JB.187.1.202-212.2005 15601704 PMC538824

[B79] Miyake T Shiba T Kameda A Ihara Y Munekata M Ishige K Noguchi T The gene for an exopolyphosphatase of Pseudomonas aeruginosa DNA Res 1999 6 103 108 10.1093/dnares/6.2.103 10382967

[B80] Kim HY Schlictman D Shankar S Xie Z Chakrabarty AM Kornberg A Alginate, inorganic polyphosphate, GTP and ppGpp synthesis co-regulated in Pseudomonas aeruginosa: implications for stationary phase survival and synthesis of RNA/DNA precursors Mol Microbiol 1998 27 717 725 10.1046/j.1365-2958.1998.00702.x 9515698

[B81] Brown MR Kornberg A Inorganic polyphosphate in the origin and survival of species Proc Natl Acad Sci USA 2004 101 16085 16087 10.1073/pnas.0406909101 15520374 PMC528972

[B82] Ishige K Noguchi T Polyphosphate:AMP phosphotransferase and polyphosphate:ADP phosphotransferase activities of Pseudomonas aeruginosa Biochem Biophys Res Commun 2001 281 821 826 10.1006/bbrc.2001.4415 11237733

[B83] Cheek S Ginalski K Zhang H Grishin NV A comprehensive update of the sequence and structure classification of kinases BMC Struct Biol 2005 5 6 10.1186/1472-6807-5-6 15771780 PMC1079889

[B84] Lange R Fischer D Hengge-Aronis R Identification of transcriptional start sites and the role of ppGpp in the expression of rpoS, the structural gene for the sigma S subunit of RNA polymerase in Escherichia coli J Bacteriol 1995 177 4676 4680 7642494 10.1128/jb.177.16.4676-4680.1995 PMC177232

[B85] Hengge-Aronis R Survival of hunger and stress: the role of rpoS in early stationary phase gene regulation in E. coli Cell 1993 72 165 168 10.1016/0092-8674(93)90655-A 8425216

[B86] Shiba T Tsutsumi K Yano H Ihara Y Kameda A Tanaka K Takahashi H Munekata M Rao NN Kornberg A Inorganic polyphosphate and the induction of rpoS expression Proc Natl Acad Sci USA 1997 94 11210 11215 10.1073/pnas.94.21.11210 9326588 PMC23418

[B87] Loewen PC Hu B Strutinsky J Sparling R Regulation in the rpoS regulon of Escherichia coli Can J Microbiol 1998 44 707 717 9830102 10.1139/cjm-44-8-707

[B88] McCann MP Kidwell JP Matin A The putative sigma factor KatF has a central role in development of starvation-mediated general resistance in Escherichia coli J Bacteriol 1991 173 4188 4194 2061293 10.1128/jb.173.13.4188-4194.1991 PMC208069

[B89] Blum E Py B Carpousis AJ Higgins CF Polyphosphate kinase is a component of the Escherichia coli RNA degradosome Mol Microbiol 1997 26 387 398 10.1046/j.1365-2958.1997.5901947.x 9383162

[B90] Rodriguez RJ Polyphosphate present in DNA preparations from filamentous fungal species of Colletotrichum inhibits restriction endonucleases and other enzymes Anal Biochem 1993 209 291 297 10.1006/abio.1993.1122 8385889

[B91] McLaren RS Newbury SF Dance GS Causton HC Higgins CF mRNA degradation by processive 3'-5' exoribonucleases in vitro and the implications for prokaryotic mRNA decay in vivo J Mol Biol 1991 221 81 95 1920421

[B92] Maurizi MR Proteases and protein degradation in Escherichia coli Experientia 1992 48 178 201 10.1007/BF01923511 1740190

[B93] Chung CH Goldberg AL DNA stimulates ATP-dependent proteolysis and protein-dependent ATPase activity of protease La from Escherichia coli Proc Natl Acad Sci USA 1982 79 795 799 10.1073/pnas.79.3.795 6461007 PMC345839

[B94] Goldberg AL The mechanism and functions of ATP-dependent proteases in bacterial and animal cells Eur J Biochem 1992 203 9 23 10.1111/j.1432-1033.1992.tb19822.x 1730246

[B95] Kuroda A Nomura K Ohtomo R Kato J Ikeda T Takiguchi N Ohtake H Kornberg A Role of inorganic polyphosphate in promoting ribosomal protein degradation by the Lon protease in E. coli Science 2001 293 705 708 10.1126/science.1061315 11474114

[B96] Kuroda A Tanaka S Ikeda T Kato J Takiguchi N Ohtake H Inorganic polyphosphate kinase is required to stimulate protein degradation and for adaptation to amino acid starvation in Escherichia coli Proc Natl Acad Sci USA 1999 96 14264 14269 10.1073/pnas.96.25.14264 10588694 PMC24425

[B97] Miller CG Protein degradation and proteolytic modification 1996 Washington, DC: American Society for Microbiology

[B98] Smith CK Baker TA Sauer RT Lon and Clp family proteases and chaperones share homologous substrate-recognition domains Proc Natl Acad Sci USA 1999 96 6678 6682 10.1073/pnas.96.12.6678 10359771 PMC21974

[B99] Hua SS Markovitz A Regulation of galactose operon at the gal operator-promoter region in Escherichia coli K-12 J Bacteriol 1975 122 510 517 165171 10.1128/jb.122.2.510-517.1975 PMC246085

[B100] Charette MF Henderson GW Doane LL Markovitz A DNA-stimulated ATPase activity on the lon (CapR) protein J Bacteriol 1984 158 195 201 6325386 10.1128/jb.158.1.195-201.1984 PMC215398

[B101] Nomura K Kato J Takiguchi N Ohtake H Kuroda A Effects of inorganic polyphosphate on the proteolytic and DNA-binding activities of Lon in Escherichia coli J Biol Chem 2004 279 34406 34410 10.1074/jbc.M404725200 15187082

[B102] Itoh H Kawazoe Y Shiba T Enhancement of protein synthesis by an inorganic polyphosphate in an E. coli cell-free system J Microbiol Methods 2006 64 241 249 10.1016/j.mimet.2005.05.003 15979174

[B103] McInerney P Mizutani T Shiba T Inorganic polyphosphate interacts with ribosomes and promotes translation fidelity in vitro and in vivo Mol Microbiol 2006 60 438 447 10.1111/j.1365-2958.2006.05103.x 16573692

[B104] Zhao J Niu W Yao J Mohr S Marcotte EM Lambowitz AM Group II intron protein localization and insertion sites are affected by polyphosphate PLoS Biol 2008 6 e150 10.1371/journal.pbio.0060150 18593213 PMC2435150

[B105] Kornberg SR Adenosine triphosphate synthesis from polyphosphate by an enzyme from Escherichia coli Biochim Biophys Acta 1957 26 294 300 10.1016/0006-3002(57)90008-2 13499364

[B106] Butler L A suggested approach to ATP regeneration for enzyme technology applications Biotechnol Bioeng 1977 19 591 593 10.1002/bit.260190415 322741

[B107] Hoffman RC Jr Wyman PL Smith LE Nolt CL Conley JL Hevel JM Warren JP Reiner GA Moe OA Jr Immobilized polyphosphate kinase: preparation, properties, and potential for use in adenosine 5'-triphosphate regeneration Biotechnol Appl Biochem 1988 10 107 117 2838045

[B108] Liu Z Zhang J Chen X Wang PG Combined biosynthetic pathway for de novo production of UDP-galactose: catalysis with multiple enzymes immobilized on agarose beads Chembiochem 2002 3 348 355 10.1002/1439-7633(20020402)3:4<348::AID-CBIC348>3.0.CO;2-K 11933236

[B109] Nahalka J Gemeiner P Bucko M Wang PG Bioenergy beads: a tool for regeneration of ATP/NTP in biocatalytic synthesis Artif Cells Blood Substit Immobil Biotechnol 2006 34 515 521 10.1080/10731190600862886 16893814

[B110] Zhao H van der Donk WA Regeneration of cofactors for use in biocatalysis Curr Opin Biotechnol 2003 14 583 589 10.1016/j.copbio.2003.09.007 14662386

[B111] Kondo H Tomioka I Nakajima H Imahori K Construction of a system for the regeneration of adenosine 5'-triphosphate, which supplies energy to bioreactor J Appl Biochem 1984 6 29 38 6490579

[B112] Crans DC Kazlauskas RJ Hirschbein BL Wong CH Abril O Whitesides GM Enzymatic regeneration of adenosine 5'-triphosphate: acetyl phosphate, phosphoenolpyruvate, methoxycarbonyl phosphate, dihydroxyacetone phosphate, 5-phospho-alpha-D-ribosyl pyrophosphate, uridine-5'-diphosphoglucose Methods Enzymol 1987 136 263 280 2446104 10.1016/s0076-6879(87)36027-6

[B113] Shih YH Whitesides GM Large-scale ATP-requiring enzymatic phosphorylation of creatine can be driven by enzymatic ATP regeneration J Org Chem 1977 42 4165 4166 10.1021/jo00445a046 925785

[B114] Kim DH Kang SY Seo WM Shim SH Yang JY Woo JS Jang KS Kim BG Sohng JK Synthesis of sialyl-vancomycin and derivatives International Conference on Biology and Chemistry of Sialic Acids; 21-26 July 2008; Moscow, St. Peterburg 2008 American Society for Microbiology 96

[B115] Iwamoto S Motomura K Shinoda Y Urata M Kato J Takiguchi N Ohtake H Hirota R Kuroda A Use of an Escherichia coli recombinant producing thermostable polyphosphate kinase as an ATP regenerator to produce fructose 1,6-diphosphate Appl Environ Microbiol 2007 73 5676 5678 10.1128/AEM.00278-07 17616610 PMC2042086

[B116] Sato M Masuda Y Kirimura K Kino K Thermostable ATP regeneration system using polyphosphate kinase from Thermosynechococcus elongatus BP-1 for D-amino acid dipeptide synthesis J Biosci Bioeng 2007 103 179 184 10.1263/jbb.103.179 17368402

[B117] Benner SA Sismour AM Synthetic biology Nat Rev Genet 2005 6 533 543 15995697 10.1038/nrg1637 PMC7097405

[B118] Jewett MC Forster AC Update on designing and building minimal cells Curr Opin Biotechnol 2010 21 697 703 10.1016/j.copbio.2010.06.008 20638265 PMC2952674

[B119] Gibson DG Glass JI Lartigue C Noskov VN Chuang RY Algire MA Benders GA Montague MG Ma L Moodie MM Merryman C Vashee S Krishnakumar R Assad-Garcia N Andrews-Pfannkoch C Denisova EA Young L Qi ZQ Segall-Shapiro TH Calvey CH Parmar PP Hutchison CA Smith HO Venter JC Creation of a bacterial cell controlled by a chemically synthesized genome Science 2010 329 52 56 10.1126/science.1190719 20488990

[B120] Sagan L On the origin of mitosing cells J Theor Biol 1967 14 255 274 11541392 10.1016/0022-5193(67)90079-3

[B121] Kimura M Recent development of the neutral theory viewed from the Wrightian tradition of theoretical population genetics Proc Natl Acad Sci USA 1991 88 5969 5973 10.1073/pnas.88.14.5969 2068072 PMC52003

[B122] Nosenko T Bhattacharya D Horizontal gene transfer in chromalveolates BMC Evol Biol 2007 7 173 10.1186/1471-2148-7-173 17894863 PMC2064935

[B123] Ng WL Bassler BL Bacterial quorum-sensing network architectures Annu Rev Genet 2009 43 197 222 10.1146/annurev-genet-102108-134304 19686078 PMC4313539

